# Combining a QSAR Approach and Structural Analysis to Derive an SAR Map of Lyn Kinase Inhibition

**DOI:** 10.3390/molecules23123271

**Published:** 2018-12-11

**Authors:** Imane Naboulsi, Aziz Aboulmouhajir, Lamfeddal Kouisni, Faouzi Bekkaoui, Abdelaziz Yasri

**Affiliations:** 1 AgroBioSciences Research Division, Mohammed VI Polytechnic University, Lot 660–Hay Moulay Rachid, 43150 Ben-Guerir, Morocco; imane.naboulsi@um6p.ma (I.N.); lamfeddal.kouisni@um6p.ma (L.K.); Faouzi.Bekkaoui@um6p.ma (F.B.); 2 Organic Synthesis, Extraction and Valorization Laboratory, Faculty of Sciences Ain Chock, Hassan II University, Km 8 El Jadida Road, 20100 Casablanca, Morocco; aboulmouhajir@gmail.com; 3 Team of Molecular Modeling and Spectroscopy, Faculty of Sciences, Chouaib Doukkali University, 24000 El Jadida, Morocco; 4 School of Agriculture, Fertilizer and Environment Sciences, Mohammed VI Polytechnic University, Lot 660 Hay Moulay Rachid, 43150 Ben Guerir, Morocco

**Keywords:** Lyn kinase, inhibitors, QSAR, ANN, GLM, SAR

## Abstract

Lyn kinase, a member of the Src family of protein tyrosine kinases, is mainly expressed by various hematopoietic cells, neural and adipose tissues. Abnormal Lyn kinase regulation causes various diseases such as cancers. Thus, Lyn represents, a potential target to develop new antitumor drugs. In the present study, using 176 molecules (123 training set molecules and 53 test set molecules) known by their inhibitory activities (IC_50_) against Lyn kinase, we constructed predictive models by linking their physico-chemical parameters (descriptors) to their biological activity. The models were derived using two different methods: the generalized linear model (GLM) and the artificial neural network (ANN). The ANN Model provided the best prediction precisions with a Square Correlation coefficient R^2^ = 0.92 and a Root of the Mean Square Error RMSE = 0.29. It was able to extrapolate to the test set successfully (R^2^ = 0.91 and RMSE = 0.33). In a second step, we have analyzed the used descriptors within the models as well as the structural features of the molecules in the training set. This analysis resulted in a transparent and informative SAR map that can be very useful for medicinal chemists to design new Lyn kinase inhibitors.

## 1. Introduction

Many signaling pathways transmit extracellular signals by altering the phosphorylation state of tyrosine residues. Phosphorylation of proteins in which tyrosine amino acid residue is phosphorylated by tyrosine kinases by the addition of a covalently bound phosphate group of ATP (adenosine triphosphate) [1], accounts only 0.1% of total protein phosphorylation in mammals. However, tyrosine kinases play a key role in the regulation of many biological phenomena such as cell proliferation, differentiation and motility. There are two families of tyrosine kinases: receptor tyrosine kinases (RTK) and non-receptor tyrosine kinases (NRTK) [2].

The existence of multiple conformations of kinases (active and non-active state) and the structural diversity of the ATP-binding site as well as the activation loop provide different strategies for designing inhibitors. Some inhibitors, by binding into the active site of the receptor tyrosine kinase, block the signal transduction resulting from the binding of certain growth factors (EGF, FGF, Gas6…) to their receptors (EGFR, FGFR, AXL …) and consequently the growth factor activity. These inhibitors are often used to prevent the tumor’s growth because many cellular tyrosine kinases are produced by the proto-oncogene and they are the most frequent oncogenesis mechanism in human cancer [1,3,4].

One of the most important and the largest non-receptor tyrosine kinases family is the Src family. It is considered for targeted therapies because Src family members are essential intermediaries in signal transduction and they can interact with a variety of growth factors, proliferating factors, and regulators of gene expression (migration, adhesion, differentiation, angiogenesis, invasion, immune function and G-protein-coupled receptors) [3,5,6]. The Src family of tyrosine kinases comprises 11 related kinases: Blk, Fgr, Fyn, Hck, Lck, Lyn, c-Src, c-Yes, Yrk, Frk (also known as Rak) and Srm with specific functions and domains. Some members of these kinases are exclusively present in certain cells as breast, colon, lung, hematopoietic, adipocyte, hepatocyte, lymphoid cells, as well as in skeleton cells [6,7].

Src signaling pathways are among the leading causes of cancer, and Src inhibitors are the keys of stopping many tumorigeneses. Therefore, that is why most of the FDA-approved protein kinase inhibitors are directed against the activation of many Src family tyrosine kinases (STKs) pathways including cell division and survival [7,8].

Lyn non-receptor tyrosine kinase is a member of Src family [9]. Lyn kinase plays an important role in the regulation of a variety of epithelial and hematopoietic cells, including the regulation of innate and adaptive immune responses, hematopoiesis, responses to growth factors and cytokines, integrin signaling, responses to DNA and genotoxic agents, as well as drug resistance [9,10,11]. This tyrosine kinase is a critical regulator of several cellular processes of many human cancer cells. The over-expression of Lyn gene according to various studies is highly correlated with the development and progression of several tumors as esophageal adenocarcinoma [12], prostate cancer (Castrate-resistant prostate cancer) [13,14], pancreatic cancer [15], cervical cancer [16], breast cancer [17,18], and it can be the cause of hepatic fibrosis [19].

Some studies have proven that Lyn is overactive in the hematological malignancies including chronic myelogenous leukemia, chronic lymphocytic leukemia B [20], Burkitt lymphoma [21], and the most common cancer diagnosed in children, Acute Lymphoblastic Leukemia (ALL) [22]. It has also been shown that the inhibition of lyn is a promoter treatment of lymphoma resistance [23,24]. Lyn is also involved in nilotinib resistance to cancer treatments [25,26], Zardan et al. suggested Lyn as a critical regulator of androgen receptor (AR) expression and activity, particularly in androgen-deprived conditions [14]. He et al. found that Lyn plays an important role in the development and progression of glioblastoma, the most aggressive brain tumors [27]. Developing new Lyn kinase inhibitors is an important therapeutic approach to block diseases where Lyn is heavily involved.

In the last decades, the identification and development of new drugs, medicinal chemists have benefited from drug rational design thanks to the chemoinformatics and molecular modeling approaches. Quantitative Structure-Activity Relationships (QSAR) is one of the chemoinformatics methodologies that allows medicinal chemist to correlate variations in a biological response of a ligand to its structural variations.

QSAR is a helpful methodology used in these recent years in drug discovery research [28,29,30]. In QSAR, the central idea is to link, through a mathematical function, several properties or molecular descriptors (topological, electronic, physico-chemical parameters…) to the activity of a set of molecules [31]. The obtained relationship is materialized by a mathematical model that can be used to predict the activity of new or existing molecules when their structural properties are known. These predictions can be also used to prioritize the organic synthesis of a small set of potentially active molecules. However, QSAR approach suffers from the fact that the predictive models are sometimes very difficult to use (qualified as black-boxes) directly during the design by medicinal chemists whose main objective is to establish the structure-activity relationships (SAR) map of the molecules under investigation [32]. It is also hard to use such models to provide to chemists future directions for modifying the molecules to improve a biological property of interest.

In the present study, using known ligands and their inhibitory activities against Lyn kinase, we constructed and validated predictive models using QSAR approach. We have also analyzed the selected molecular descriptors and the structural fragments of the inhibitors to draw a SAR map for the inhibition of Lyn kinase that can be used to build new and potentially active inhibitors.

## 2. Materials and Methods

### 2.1. Dataset Source and Preparation

A set of 440 molecules with their two-dimensional atomic coordinates and IC_50_ were fetched from the BindingDB database [33]. This set was reduced to 176 molecules by applying a set of filters: (1) Lipinski’s rule (number of hydrogen-bond donors less than 5, number of hydrogen acceptor less than 10, molecular weight less than 500 and log P less than 5; and the sum of donors and acceptors (N + O) less than 10) [34], (2) filtering out duplicates (we kept only the molecule that has the highest IC_50_) and (3) removing all the molecules without reported IC_50_ for Lyn kinase.

We randomly distributed the 176 molecules into two subsets: 123 molecules (70%) represent the training set to derive and validate internally the models and 53 molecules (30%) for the test set, to perform external validation and assessment of its extrapolation capacity to new data (Supplementary Materials). The targeted biological property of our QSAR study is IC_50_ (concentration of the ligand that induces 50% of the inhibition of the enzyme activity). The IC_50_ values have been converted to molar units pIC_50_ (defined as −log_10_ IC_50_). The distributions of pIC_50_ values (max = 4.34; min = 9.30) within the training and test set reproduced their distributions within the whole set.

### 2.2. Calculation of Molecular Descriptors

A set of 184 two-dimensional molecular descriptors were calculated by Molecular Operating Environment (MOE) package (version 2008.10, Chemical Computing Group, Montreal, Canada) [35]. These descriptors cover different classes of molecular parameters such as chemical constitution, topology, geometry and electrostatic properties, wave function, potential energy surface or some combination of these items for a given chemical structure.

### 2.3. Diversity Analysis

The structural diversity of the data was defined by using Principal Component Analysis (PCA) which is a powerful approach for exploring high-dimensional data [36]. We calculated the principles components (PC) using JMP (14.0.1) package [37] for a data matrix *p* × *n* dimension where *n* = 176 inhibitors and *p* = 184 descriptors.

### 2.4. Descriptors Selection

The 184 molecular structural descriptors for the 123 inhibitors of the training set data have been reduced sequentially using two phases: (1) We first used variable importance calculated from Partial Least-Squares (PLS) method where we excluded all the descriptors that have a Variable Importance less than 1, (2) in a second step, the resulting descriptors were submitted to a stepwise forward selection.

### 2.5. Model Development and Validation

To build our models, we used a training set of 172 molecules selected randomly from the initial data set. To fit the physico-chemical properties of the training set to the pIC_50_ values, we used Generalized Linear Model (GLM) as a linear discriminant analysis method [38] and Artificial Neuronal Network (ANN), with feedforward backpropagation to train the model, as a nonlinear method [29,39]. Both methods are implemented in JMP software package [37]. For the GLM, we generated our models using a normal distribution, a unitary link function, and Maximum likelihood as estimation method. For ANN models, we varied the hidden layer size from range 3 to 12 neurons and used 10-fold cross-validation repeated 10 times as an internal validation method process to validate the models. For the external validation of the model, we used a test set containing 53 molecules selected randomly from the initial data set.

### 2.6. Domain of Applicability of the Models

To assess the reliability of the QSAR model for prediction purposes, we defined a domain of its applicability using a Mahalanobis distance-based approach [40]. The Mahalanobis distance to the training set is calculated for each molecule to be predicted. This distance, compared to Euclidian distance, accounts for the covariance among variables [40]. We have implemented a python program implementing the Mahalanobis distance algorithm as defined below.

In general, if $\vec{x}$ = [x_1_, x_2_,…, x_p_]^T^ and $\vec{µ}$ = [µ_1_, µ_2_,…, µ_i_]^T^ are multivariate data-observations drawn from a set of *p* variables with a *p* × *i* covariance matrix C, then the Mahalanobis distance DM between them is defined as:

(1) $${\mathrm{DM}}^{2}={\left(x-\mu \right)}^{T}\times C^{-1}\times \left(x-\mu \right)$$

where ${\mathrm{DM}}^{2}$ = Mahalanobis distance; x = Vector of data; μ = Vector of mean values of independent variables; C^−1^ = Inverse Covariance matrix of independent variables and T = Transposed matrix.

## 3. Results and Discussion

### 3.1. Diversity Analysis

**Figure 1:**
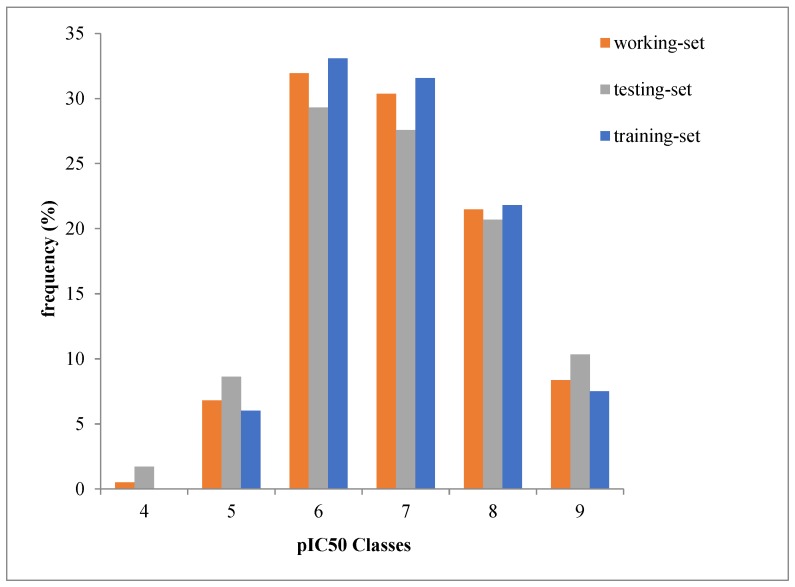
pIC_50_ values distribution in the training and test sets.

During the split of initial data (all data set) into training set of 123 molecules and a test set of 53 molecules, we ensured that the distribution of pIC_50_ value remains the same in the training and test sets as in the initial data set (Figure 1).

**Figure 2:**
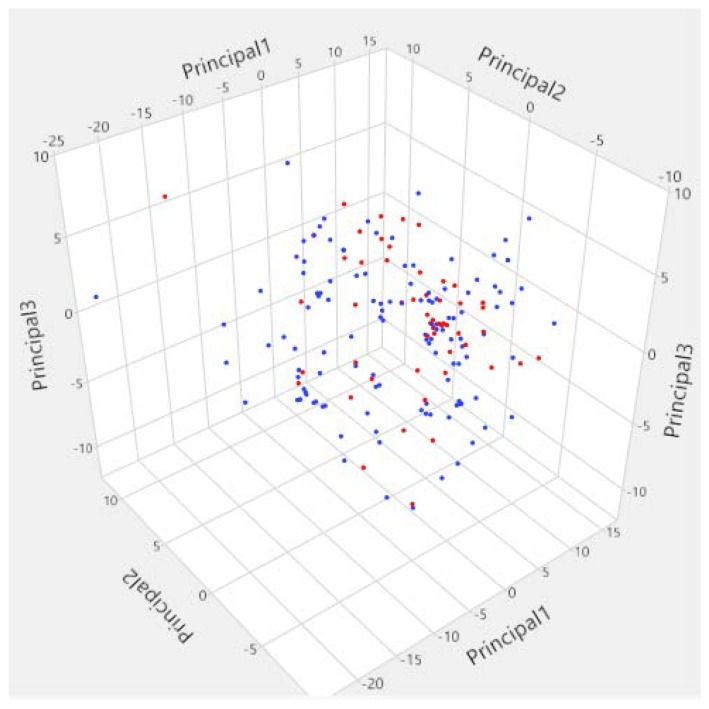
Visualization of descriptors space of Lyn tyrosine kinase inhibitors using principal component analysis (blue points = Training set; red points = test set).

The PCA analysis of the molecular descriptors space explained 56.34% of the global information of the original space (PC1: 35.4%; PC2: 12.8% and PC3: 8.14%). This analysis showed that the molecules in the training set and the test set were distributed homogeneously in the PCA space resulting in a good structural diversity in the data (Figure 2). This is in agreement with the different chemotypes represented in the initial data as shown in Table 1.

### 3.2. Descriptors Pertinence

The initial descriptor pool number (184 descriptors) was first reduced by eliminating out the descriptors with constant and near constant values. PLS was then used to further reduce the number of descriptors according to variable importance in the model. In fact, the PLS model resulted in a coefficient of determination R^2^ of 0.72 and a cross-validated coefficient of determination q^2^ of 0.63. When the variable importance threshold was set to the unit value, only 80 descriptors were retained. After using a stepwise forward selection procedure, the set of descriptors was further reduced to 35 descriptors that were then subjected to the data modeling step with the aim to find the best fit between the descriptors and the inhibitory activities of the molecules. These descriptors account for 7 different molecular categories as defined in Table 2.

The selected descriptors cover the main structural features of the molecules needed for their biological activity. In fact, the physico-chemical properties such as logP, logS, MR, apol TPSA, logP and Subdivided Surface Areas represent molecular features that could explain the bioavailablity of the drugs. Pharmacophoric features, connectivity and shape indices as well as partial charge properties are features that represent the mode of interaction of drugs with their targeted receptor. Finally, atom and bond accounts and adjacency and distance matrix descriptors are features representing the topology as well as the geometry of the molecules.

### 3.3. QSAR Model Derivation and Validation

Using GLM approach to fit the 35 selected descriptors to the pIC_50_ values of the training set resulted in a weak predictive power as judged by the correlation coefficient between experimental and predicted values of R^2^ = 0.65 and a Mean Square Error RMSE = 0.64. When the model is applied to the test set, the correlation coefficient drops down to a value of R^2^ = 0.39 and RMSE = 0.85. Consequently, GLM was not able to provide neither a predictive model for the molecules in the training set for the inhibition of Lyn kinase nor an extrapolation power to molecules used in the test set. The GLM model was not capable of predicting the pIC_50_ value of the Lyn kinase inhibitors even if the descriptor selection step was done using the statistical procedure “stepwise forward selection procedure”. This is due to the fact that the stepwise forward selection procedure uses multiple linear regression method to score the selected set of descriptors and it is not intended to derive a robust predictive model. Again, when used with the GLM, the combination of the descriptors in a linear way did not results in a predictive QSAR model.

**Figure 3:**
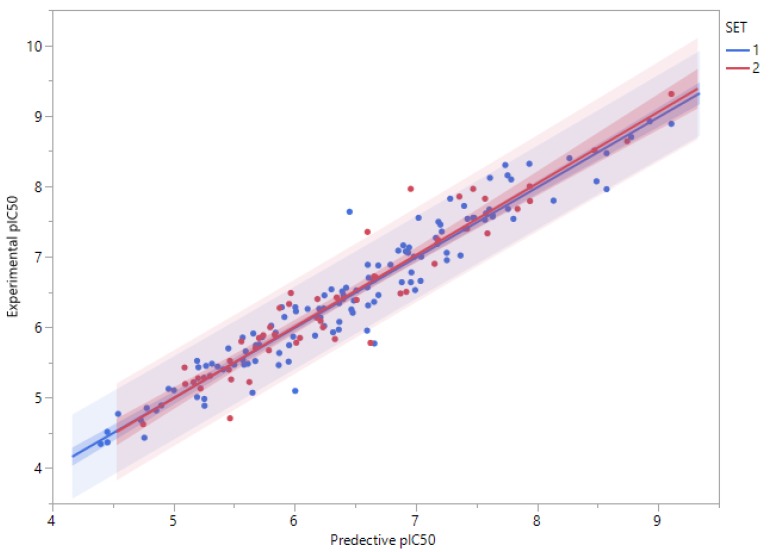
Correlation plots between predicted and experimental pIC_50_ values for the training and the test sets derived using ANN methods. Training set (SET 1) molecules are represented by blue points and test set molecules (SET 2) are represented by red points.

When ANN approach was used, the derived model showed good predictive performance for the training set and good extrapolation to new and unseen molecules of the test set. In fact, several ANN models were built by varying the size of the hidden layer by increasing the number of neurons from 3 to 12 (Table 3). The predictive capacity of the model increased with the size of the hidden layer and reached a plateau when the number of neurons exceeded the value of 9. The model using 9 neurons in the hidden layer presented the best fit and the best cross-validated results as judged by the cross-validated correlation coefficient and the root mean squared error (R_T_^2^ = 0.92, RMSE_T_ = 0.29, R_v_^2^ = 0.90 and RMSE_V_ = 0.32). This model was applied to predict the molecules in the test set (Table 4) and resulted in a very good correlation coefficient between the experimental and predicted values of pIC_50_ and root mean-squared error (R_Ts_^2^ = 0.91 and RMSE_Ts_ = 0.33) (Figure 3).

The model derivation and validation step resulted in a very good QSAR model using the ANN approach while the GLM approach was not able to derive useful models. This is explained by the fact that the training set contains high structural molecular diversity and high nonlinear underlying relationships between the structural variations and the biological activities of the models that only a nonlinear approach as ANN was able to conceptualize.

### 3.4. Applicability Domains of QSAR Models

**Figure 4:**
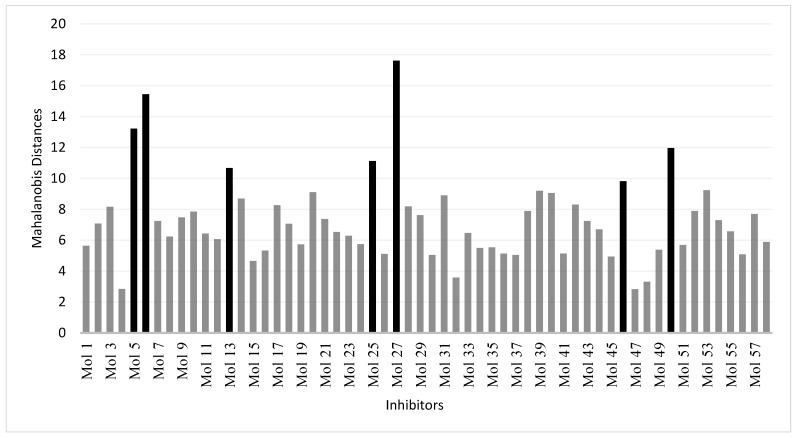
Calculated Mahalanobis distances of the molecules in the test set.

To define the domain of applicability of the derived and validated QSAR model, we have used the Mahalanobis distance as a distance-based metric approach. This method calculates a distance between each molecule to be predicted (molecules in the test set) and the closest molecule in the training set. Any molecule above a threshold distance is considered to be unpredictable by the model or predictable with low confidence. When applied to the training set, the most distant molecule of the rest of the molecules is at a Mahalonbis distance of 9. When using a threshold value of 9, only seven molecules in the test set were distant from the training set (Figure 4). This analysis showed that most of the test set molecules can be safely predicted by the model as judged by the Mahalanobis distance.

### 3.5. Structure-Activity Relationship Map Derivation

**Figure 5:**
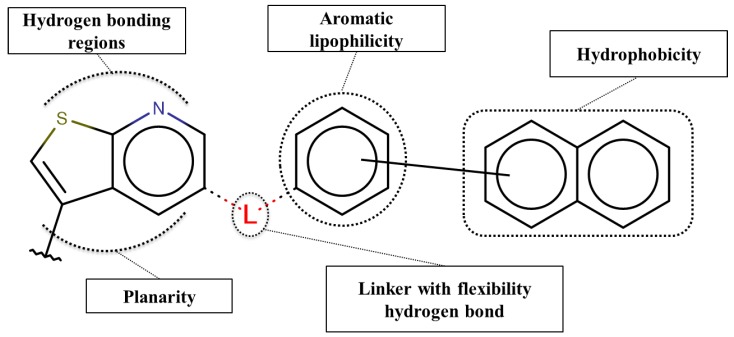
SAR maps derived from the analysis of the physico-chemical descriptors and the structural features of the molecules in the training set.

Based on our selection of the most pertinent descriptors used in the QSAR model and the structural analysis of the molecules in the training set Table 1), we tried to derive a SAR map that explains Lyn kinase inhibition and also the predctions from the selected descriptors used in the QSAR model (Figure 5). Indeed, most of the active molecules in the training set hold in one of their extrimities a planar bicyclic aromatic system that can be heterocyclic or not. This feature is represented by the number of double bond descriptor (b_count) correlated to aromatic planar rings and the number of donors and acceptors of hydrogen bonds (a_acc, lip_acc, a_don, lip_don) which lead to heterocyclic rings. Another common structural element in the active molecules is a central aromatic ring wich can again be reprensented by the number of the double bonds descriptor. This part of the molecule is usualy linked to the plan bicyclic system by a flexible linker. The flexibility is encoded in the kier_flex descriptor. A third common strutural element of the active molecules is an aromatic ring system localized at the other extrimity of the molecule. The three aromatic rings (planar heterocyle, central aromatic ring and an aromatic system being opposite of the first aromatic system) can be also encoded by the lipophilicity descripor (logP). With all these aromatic rings, the majority of active molecules present a high molecular volume that is encoded in the molar refractivity descripto (MR). Finnaly, the heterocyclic ring system as well as the number of donors and acceptors of hydrogen bonds are at the origin of the polarizability of the molecule which is encoded in the polar surface area descriptos (TPSA, apol and PEOPE).

Overall, considering the common structural features and some of the selected and used descriptors in the QSAR model, we could suggest a SAR map for the inhibition of the Lyn kinase as follows: (1) a planar and heterocyclic ring system that holds hydrogen bond donnors and acceptors, (2) a Linker to keep the flexibility of the molecule, (3) an hydrophobic and aromatic central part, (4) a lipophilic and aromatic ring system.

The derived SAR map can be found when analysing the structure of some published Lyn kinase inhibitiors. Indeed, a Lyn kinase inhibiotors (INNO-406, Nilotinib), with IC_50_ of 220 nM, was reported in the work of Horio et al. [42]. This compound bears a pyridinyl group as hydrogen bonding region, an amino group as a linker and a central substitued benzyl group as the hydrophobic region. Kim et al. obtained a Lyn kinase inhibitor (PCI-32765) [43], with an IC_50_ of 200 nM, showing an aminopyrimidine moity playing the role of the hydrogen bonding region, a benzyl group as the aromatic moity linked to another benzyl group presenting the hydrophobic region. In the work of Goldberg et al. a reported Lyn kinase inhibitor (BDBM50218682), with an IC_50_ of 230 nM, presented an aminopyridin moity as the hydrogen bonding region, an amid bond as the linker, a central aromatic fused cycle, and a substitued benzamid part playing the role of the hydrophobic region [44].

## 4. Conclusions

Several physicochemical, topological and electronic molecular descriptors combined with a GLM method and ANN method were used for modeling and predicting Lyn kinase inhibitors. The best model was determined based on the values of correlation coeficcient between the experimental and the predicted pIC_50_ values for the training set and the test set. The ANN model obtained the highest prediction performance. The selected descriptors involved in the ANN model as well as the structural features of the training set were analyzed together to draw an informative SAR map that was in good agreement with published Lyn kinase inhibitors.

Overall, this study demonstrates that the machine learning method combined to molecular parameters can be used for in silico prediction of Lyn kinase inhibition and that the selected descriptors together with structural features derived from the training molecules can serve to build an SAR map to explain Lyn kinase inhibition.

## Figures and Tables

**Table 1:** Representative chemical series of inhibitors of Lyn tyrosine kinase.

Compounds	
38 Molecules	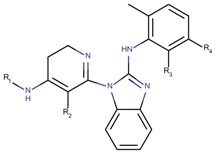
33 Molecules	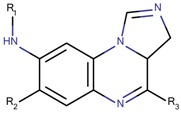
32 Molecules	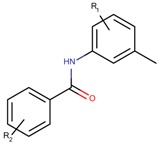
12 Molecules	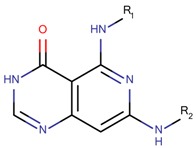
12 Molecules	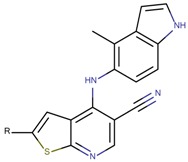
26 Molecules	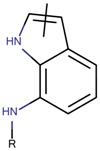

**Table 2:** Categories and definitions of computed molecular descriptors [41].

	Categories of Descriptors	Definition	Categories of Descriptors	Definition
**Physico-Chemical Properties**	LogP (o/w)	Log of the octanol/water partition coefficient (including implicit hydrogens)	MR	Molecular refractivity (including implicit hydrogens)
	logS	Log of the aqueous solubility (mol/L)	TPSA	Polar surface area (Å2) calculated using group contributions to approximate the polar surface area from connection table information only
	apol	Sum of the atomic polarizabilities (including implicit hydrogens)	SlogP_VSA0 SlogP_VSA1 SlogP_VSA3 SlogP_VSA5 SlogP_VSA6	Subdivided logP Surface Areas are descriptors based on an approximate accessible van der Waals surface area (in Å2) calculation for each atom along with its contribution to logP property
**Atom and Bond Counts**	PEOE_RPC_−	Relative negative/positive partial charge: the smallest negative qi divided by the sum of the negative qi. Q_RPC−/Q_RPC+ is identical to RPC−/RPC+ which has been retained for compatibility	PEOE_VSA_0	Sum of *vi* (a der Waals surface area of atom i) where *qi* (partial charge of atom i) is in the range (−0.05, 0.00)
	PEOE_RPC_+		PEOE_VSA_FPOS	Fractional positive van der Waals surface area. This is the sum of the vi such that *qiis* non-negative divided by the total surface area. The vi are calculated using a connection table approximation
**Atom and Bond Counts**	b_double	Number of double bonds. Aromatic bonds are not considered to be double bonds	lip_acc	The number of O and N atoms
	a_ICM	Atom information content (mean). This is the entropy of the element distribution in the molecule (including implicit hydrogens but not lone pair pseudo-atoms)	lip_don	The number of OH and NH atoms
	b_count	Number of bonds (including implicit hydrogens)	lip_druglike	One if and only if Lipinski’s rules violation < 2 otherwise zero
**Pharmacophoric Features**	a_acc,	Number of hydrogen bond acceptor atoms (not counting acidic atoms but counting atoms that are both hydrogen bond donors and acceptors such as -OH)	vsa_don,	Approximation to the sum of VDW surface areas of pure hydrogen bond donors (not counting basic atoms and atoms that are both hydrogen bond donors and acceptors such as -OH) (Å2)
	a_don,	Number of hydrogen bond donor atoms (not counting basic atoms but counting atoms that are both hydrogen bond donors and acceptors such as -OH)	vsa_other	Approximation to the sum of VDW surface areas (Å2) of atoms typed as “other”
**Connectivity and Shape Indices**	chi0	Atomic connectivity index (order 0)	KierFlex	Kier molecular flexibility index
	chil_C	Carbon connectivity index (order 1)		
**Adjacency and Distance Matrix Descriptors**	VDistMa	If m is the sum of the distance matrix entries	BCUT_SLOGP_1	The BCUT descriptors using atomic contribution to logP (using the Wildman and Crippen SlogP method)
	WeinerPath	Wiener path number.	BCUT_SLOGP_3	
	balabanJ	Balaban’s connectivity topological index	GCUT_SMR_1	The GCUT descriptors using atomic contribution to molar refractivity (using the Wildman and Crippen SMR method) instead of partial charge
	BCUT_PEOE_3	Adjacency and distance matrix descriptors. The BCUT descriptors are calculated from the eigenvalues of a modified adjacency matrix	GCUT_SMR_3	
	BCUT_SMR_2	The BCUT descriptors using atomic contribution to molar refractivity (using the Wildman and Crippen SMR method) instead of partial charge		

**Table 3:** Predictive and extrapolation powers of the QSAR models derived by ANN approach.

	R_T_^2^	RMSE_T_	R_v_^2^	RMSE_V_
ANN 3-layers	0.75	0.54	0.48	0.68
ANN 4-layers	0.81	0.47	0.71	0.51
ANN 5-layers	0.76	0.53	0.64	0.56
ANN 6-layers	0.84	0.43	0.78	0.46
ANN 7-layers	0.90	0.34	0.85	0.39
ANN 8-layers	0.86	0.40	0.62	0.66
ANN 9-layers	0.92	0.29	0.90	0.32
ANN 10-layers	0.90	0.34	0.78	0.47
ANN 11-layers	0.91	0.32	0.72	0.62
ANN 12-layers	0.88	0.37	0.82	0.40
ANN 13-layers	0.86	0.40	0.74	0.51

**Table 4:** Test-set values by using ANN approach.

	R_Ts_^2^	RMSE_Ts_
ANN 3-layers	0.77	0.52
ANN 4-layers	0.82	0.46
ANN 5-layers	0.73	0.56
ANN 6-layers	0.79	0.50
ANN 7-layers	0.89	0.37
ANN 8-layers	0.87	0.40
ANN 9-layers	0.91	0.33
ANN 10-layers	0.89	0.36
ANN 11-layers	0.92	0.31
ANN 12-layers	0.91	0.33
ANN 13-layers	0.90	0.35

## Supplementary Materials

The following are available online.
