# Supplementary material for: Combining a QSAR Approach and Structural Analysis to Derive an SAR Map of Lyn Kinase Inhibition
Source: Molecules. 2018 Dec 11;23(12):3271. doi: 10.3390/molecules23123271 (PMC6320833; doi:10.3390/molecules23123271)
Supplement: Supplementary file 1 [file molecules-23-03271-s001.pdf]

Supplementary materials

Smile code	Experimental pIC50 (M)	Predicted pIC50 (M)
CN(C)C1CN(C1)CC2=C(C=C(C=C2)C(O)NC3=CC=C(C)C(=C3)NC4=NC(=CC=N4)C5=CN=CN=C5)C(F)(F)	7,54	7,66
CC1=CC2=C[NH]N=C2C=C1C3=CC=CC4=C(N)C(=NN=C34)C(N)=O	7,27	7,20
CCN1CCN(CC1)C2=CC=C(NC3=NC=NC(=C3)N(C)C(=O)NC4=C(Cl)C(=CC(=C4Cl)OC)OC)C=C2	6,52	6,40
CN(C)C/C=C/C(=O)N(C)C1=C(Cl)C=C2N=C(NC3=CC(=CC=C3C)F)C4=CN=C[N]4C2=C1	5,52	5,04
OCCNC(=O)C1=CC2=C(NC3=CC=C(OC4=CC=CC5=C4C=CS5)C(=C3)Cl)N=CN=C2NCC1	5,01	5,24
CN1CCN(CC1)C2=CC(=CC(=C2)C(F)(F)F)C(=O)NC3=CC=C(C)C(=C3)NC(=O)C4=C(C)ON=C4	7,18	6,05
NC1COCCC1NC2=CC3=C(C(=O)NC=C3)C(=N2)NC4=CC=CC5=C4[NH]C=C5	5,92	5,94
CC1=CC=C(NC2=NC(=NC=C2C(N)=O)NC3CCOCC3N)C=C1	5,40	5,60
NC1=C2C(=NC=N1)[N](N=C2C3=CC=C(OC4=CC=CC=C4)C=C3)C5CCC(CC5)NC(=O)C=C	8,92	8,67
CC1=C[NH]C2=C1C=CC=C2NC3=NC(=CC4=C3C(=O)NC=N4)NC5CCOCC5N	7,16	7,05
CC1=C(NC2=NC3=CC=CC=C3[N]2C4=NC=NC(=C4C)N)C=C(C=C1)C(=O)NC5=CC(=CC=C5)C(F)(F)F	5,10	5,87
COC1=CC(=CC=C1NC(=O)C2=CC3=CC=CC=C3[N]2C)C4=CSC5=C(\C=C\CNC6CCC(N)CC6)C=NC(=C45)N	4,89	4,89
CC(C)(C(=O)NCC[N]1C=CC2=NC=NC(=C12)NC3=CC(=C(OC4=CC=CC(=C4)Cl)C=C3)Cl)[S](C)(=O)=O	5,48	5,38
NC1CCCCC1NC2=CC3=C(C(=O)NC=N3)C(=N2)NC4=CC=CC5=C4[NH]C=C5	6,95	7,33
CC(C)C1=CC(=CC=C1)NC(=O)C2=CC(=C(C)C=C2)NC3=NC4=CC=CC=C4[N]3C5=NC=NC(=C5)N	7,82	6,65
CN1CCN(CC1)CC2=C(C=C(C=C2)C(=O)NC3=CC=C(C)C(=C3)NC4=NC(=CC=N4)C5=CN=CC=C5)C(F)(F)F	8,32	7,79
C[N]1C=C(C=N1)C2=CC(=CC=C2)C3=NC(=C(N)N=C3)C(=O)N[CH]4C5C[CH]6CC4C[C](O)(C6)C5	6,45	6,57
CC1=C(NC2=NC3=CC=CC=C3[N]2C4=NC=NC(=C4)NC5CCC(N)CC5)C=C(NC(=O)C6=CC=CC(=C6)C(F)(F)F)C=C1	6,26	6,71
CN(C)CC1=CN=CC(=C1)C2=CC3=C(S2)N=CC(=C3NC4=C(C)C5=C([NH]C=C5)C=C4)C#N	5,66	5,93
CC1=C(NC2=NC3=CC=CC=C3[N]2C4=NC=NC(=C4)NC5=CC=C(CCN6CCOCC6)C=N5)C(=CC=C1)Cl	6,67	6,41
NC1COCCC1NC2=CC3=C(C(=O)NC=N3)C(=N2)NC4=CC=CC5=C4[NH]C=C5	6,64	7,11
COC(=O)C(=O)NC1=C2CC3=C([NH]N=C3C2=CC=C1)C4=CSC(=C4)C#CCOC5=CC=CC=C5	4,51	4,31
CN1CCN(CC1)CC2=C(C=C(C=C2)C(=O)NC3=CC=C(C)C(=C3)NC4=NC(=CC=N4)C5=CN=CN=C5)C(F)(F)F	8,07	7,99
OC(=O)CN1CCN(CC1)C2=NC=CC(=C2)C3=CN=C4C=CC(=N[N]34)N5CCCC5C6=CC=CC(=C6)F	5,47	6,00

COC1=CC2=NC=NC(=C2C=C1OCCCCCCC(=O)NO)NC3=CC=CC(=C3)C#C	6,08	5,86
NC1CCCCC1NC2=NC(=C(C=N2)C(N)=O)NC3=CC(=CC=C3)[N]4N=CC=N4	6,70	6,53
CC(N)CNC1=CC2=C(C(=O)NC=N2)C(=N1)NC3=CC=CC4=C3[NH]C=C4	7,08	6,85
NC1COCCC1NC2=CC3=C(C(=O)NC=N3)C(=N2)NC4=CC=CC5=C4[NH]C=C5	6,64	6,19
CN(C)C/C=C/C(=O)N(C)C1=C(F)C=C2N=C(NC3=CC=C(F)C=C3C)C4=CN=C[N]4C2=C1	5,46	5,25
CC1=C(NC2=NC3=CC=CC=C3[N]2C4=NC=NC(=C4)NCCCCN)C=C(NC(=O)C5=CC=CC(=C5)C(F)(F)F)C=C1	6,34	6,05
CN(C)C/C=C/C(=O)N(C)C1=C(F)C=C2N=C(NC3=CC(=CC=C3C)F)C4=CN=C[N]4C2=C1	5,51	5,74
CC1=C[NH]C2=C1C=CC=C2NC3=NC(=CC4=C3C(=O)NC=C4)NC5CCCCC5N	6,26	5,92
CNC1=C(F)C=C2N=C(OC3=CC=CC=C3C)C4=CN=C[N]4C2=C1	6,89	6,70
NC1=NC=NC2=C1C(=C[N]2C3CCCC3)C4=CC=C(OC5=CC=CC=C5)C=C4	6,38	6,24
CC1=C[N](C=N1)C2=CC(=CC(=C2)F)NC(=O)C3=CC=C(C)C(=C3)NC4=NC(=CC=N4)C5=CC=CN=C5	4,82	4,90
CN1CCN(CC1)C2=CC=C(C=C2)C3=CC4=C(C(=N[N]4C)N)C(=O)N3	4,85	5,09
CN(C)C/C=C/C(=O)N(C)C1=CC=C2N=C(NC3=CC=C(O)C=C3C)C4=CN=C[N]4C2=C1	6,46	6,33
CC(OC1=NN=CC2=CC(=CC=C12)C3=C(C)C=CC(=C3)C(=O)NC4CC4)C(F)(F)F	6,02	5,98
C[N]1C=C(\C=C2/OC3=CC=CC(=C3C2=O)O)C4=C(C=CN=C14)C5=CC=C(C=C5)C(=O)NCCO	7,55	7,45
CN(C)C/C=C/C(=O)N(C)C1=CC=C2N=C(NC3=CC=C(F)C=C3C)C4=CN=C[N]4C2=C1	6,15	5,78
CC1=C(NC2=NC3=CC=CC=C3[N]2C4=NC=NC(=C4)N)C=C(C=C1)C(=O)NC5=CC=CC(=C5)C(C)(C)C	7,80	7,98
CC(OC1=CC(=CC=C1C(N)=O)C2=C(N)N=CC(=C2)C3=CC(=CS3)CN(C)C)\C=C/C(F)(F)F	5,87	5,91
CN(C)CC1=CC=CC=C1C2=CC3=C(S2)N=CC(=C3NC4=C(C)C5=C([NH]C=C5)C=C4)C#N	4,43	5,55
CC1=C[NH]C2=C1C=CC=C2NC3=NC(=CC4=C3C(=O)NC=N4)NC5CCCCC5N	7,36	7,49
COC1=C(OC)C=C2C(=CC=NC2=C1)OC3=CC4=CC=CC(=C4C=C3)C(=O)NC5CC5	7,57	7,43
COC1=C(NC(=O)C#CCN2CCCCC2)C=C3[N]4C=NC=C4C(=NC3=C1)NC5=CC=CC=C5C	7,67	7,22
CCN(CC)C/C=C/C1=C2SC=C(C3=CC=C(NC(=O)C4=CC5=CC=CC=C5[N]4C)C(=C3)OC)C2=C(N)N=C1	5,93	5,86
CN(C)C/C=C/C(=O)N(C)C1=CC=C2N=C(NC3=CC=CC=C3C)C4=CN=C[N]4C2=C1	6,21	6,42
COC1=CC(=CC=C1)NC(=O)C2=CC(=C(C)C=C2)NC3=NC4=CC=CC=C4[N]3C5=NC=NC(=C5)N	6,36	5,59
CC1=CC(=CC(=C1)NC(=O)C2=CC=C(C)C(=C2)NC3=NC(=CC=N3)C4=CC=CN=C4)[N]5C=NC(=C5)C	5,45	5,08
CC1=C(NC2=NC3=CC=CC=C3[N]2C4=NC=NC(=C4)N)C=C(NC(=O)C5=CC(=CC(=C5)Cl)Cl)C=C1	5,44	5,66

CN(C)C1CCN(C1)CC2=C(C=C(C=C2)C(=O)NC3=CC=C(C)C(=C3)NC4=NC(=CC=N4)C5=CN=CN=C5)C(F)(F)F	7,96	8,15
[O-][N+](=O)C1=CC=CC=C1C2=N[NH]C=C2/C=C/3C(=O)NC4=CC=CC=C34	8,40	8,26
CC1=C(NC2=NC3=CC=CC=C3[N]2C4=NC=NC(=C4)N)C=C(NC(=O)C5=CC(=N[NH]5)C6CC6)C=C1	7,02	6,99
CNC1CC2OC(C1OC)[N]3C4=C(C=CC=C4)C5=C6CNC(=O)C6=C7C8=C(C=CC=C8)[N]2C7=C35	7,61	8,17
COCCNC(=O)C1=CC=CC2=CC(=CC=C12)OC3=C4C=C(OC)C(=CC4=NC=C3)OC	6,56	6,34
CN1CCN(CC1)CC2=CC=C3OC(=CC3=C2)C4=C(NC5=CC=C6[NH]C=CC6=C5C)C(=CN=C4)C#N	6,14	6,27
CN1CC2CC1CN2C3=CC=C(C=C3)C4=CC=NC5=C(C6=CC=CC(=C6)O)C(=N[N]45)C7=CC=NC=C7	5,75	5,92
CN1CCN(CC1)CC2=C(F)C=C(C=C2)C(=O)NC3=CC=C(C)C(=C3)NC4=NC(=CC=N4)C5=CN=CC=C5	7,46	7,07
CC1=NC(=CC(=N1)N2CCN(CCO)CC2)NC3=CC(=NC=N3)[N]4C(=NC5=CC=CC=C45)NC6=C(C)C=CC=C6Cl	7,52	7,48
OC(=O)CN1CCN(CC1)C2=NC=CC(=C2)C3=CN=C4C=CC(=N[N]34)N5CCCC5C6=CC=CC(=C6)F	5,47	5,31
CN(C)C/C=C/C(=O)N(C)C1=C(Cl)C=C2N=C(NC3=CC=CC=C3Cl)C4=CN=C[N]4C2=C1	5,70	5,87
COC1=CC(=C(Cl)C=C1Cl)NC2=C3C=C(OC)C(=CC3=NC=C2C#N)OCCCN4CCN(C)CC4	8,10	8,36
CC1=CC=C(C=C1)[N]2N=C(C=C2NC(=O)NC3=CC=C(OCCN4CCOCC4)C5=CC=CC=C35)C(C)(C)C	6,02	6,13
CC1=C(NC2=NC3=CC=CC=C3[N]2C4=NC=NC(=C4)NCCCN)C=C(NC(=O)C5=CC=CC(=C5)C(F)(F)F)C=C1	5,95	5,88
CNC1=C(F)C=C2N=C(NC3=CC=CC=C3C)C4=CN=C[N]4C2=C1	7,72	7,42
COCC(=O)NC1=C2CC3=C([NH]N=C3C2=CC=C1)C4=CSC(=C4)C#CCOC5=CC=CC=C5	4,68	5,08
NC1CCCCC1NC2=CC3=C(C(=O)NC=C3)C(=N2)NC4=CC=CC5=C4[NH]C=C5Cl	5,74	5,76
COC1=CC=C(CN(C)C)C(=C1)C2=CC3=C(S2)N=CC(=C3NC4=C(C)C5=C([NH]C=C5)C=C4)C#N	4,89	5,15
COC1=C(CN(C)C)C=CC=C1C2=CC3=C(S2)N=CC(=C3NC4=C(C)C5=C([NH]C=C5)C=C4)C#N	5,77	6,40
OC1=CC2=C3C(=C1O)OC(=O)C4=C3C(=C(O)C(=C4)O)OC2=O	5,54	5,42
CC1=C(NC2=NC3=CC=CC=C3[N]2C4=NC=NC(=C4)Cl)C=C(NC(=O)C5=CC=CC(=C5)C(F)(F)F)C=C1	5,48	6,07
CN(C)CCN1CCN(CCC1=O)C(=O)C2=C(NC(=O)NC3=CC=CC(=C3Cl)Cl)SC(=C2)C(C)(C)C	4,37	5,97
OC1=CC(=CC=C1C2=COC=C2)CN/C=C/3C(=O)NC(=O)C4=C3C=C(I)C=C4	5,07	5,22
CC1=C(NC2=NC3=CC=CC=C3[N]2C4=NC=NC(=C4)N)C=C(NC(=O)C5=C(Cl)C=NC=C5)C=C1	6,23	6,81
CN1CCN(CC1)[S](=O)(=O)C2=CC(=CC=C2)\C=C\C3=C(NC4=CC=C5[NH]C=CC5=C4C)C(=CN=C3)C#N	6,54	6,02
CC1=C(NC2=C(C=NC3=C2C=C(S3)\C=C\C(=O)N4CCCC4)C#N)C=CC5=C1C=C[NH]5	7,55	7,47
CNC1=C(F)C=C2N=C(OC3=CC=CC=C3C)C4=CN=C[N]4C2=C1	6,89	6,79

COC1=C(C=CC=C1)C2=C(C(=O)NC2=O)C3=C[N](CCCNC(=O)OC(C)(C)C)C4=C3C=CC=N4	5,74	5,34
N#CC1=CC=C(C=C1)C2=CN=C3C=CC(=N[N]23)NCC4=CC=CC=C4	5,12	5,03
CN1CCN(CC1)C2CCC(CC2)[N]3C=C(C4=CC=C(OC5=CC=CC=C5)C=C4)C6=C3N=CN=C6N	7,54	7,55
CC1=C(NC2=C(C=NC3=C2C=C(S3)\C=C\C(=O)N4CCCC4)C#N)C=CC5=C1C=C[NH]5	7,55	7,42
COC1=CC=CC=C1C2=CC3=C(S2)N=CC(=C3NC4=C(C)C5=C([NH]C=C5)C=C4)C#N	6,51	5,91
CC1=C[N](C=N1)C2=CC(=CC=C2)NC(=O)C3=CC=C(C)C(=C3)NC4=NC(=CC=N4)C5=CC=CN=C5	4,34	4,42
CCN(CC)C/C=C/C1=C(C)C2=CC=C3N=C(NC4=C(Cl)C=CC=C4Cl)[N](C)C3=C2C(=O)N1	8,70	8,37
CC1=C(NC2=NC3=CC=CC=C3[N]2C4=NC=NC(=C4)N)C=C(NC(=O)C5=C(C=NC=C5)C(C)(C)C)C=C1	8,15	8,38
CN1CCN(CC1)CC2=CC(=N[N]2C(C)(C)C)C(=O)NC3=CC(=C(C)C=C3)NC4=NC5=CC=CC=C5[N]4C6=NC=NC(=C6)N	7,06	6,68
CN(C)C1CCN(C1)CC2=C(C=C(C=C2)C(=O)NC3=CC=C(C)C(=C3)NC4=NC(=CC=N4)C5=CN=CN=C5)C(F)(F)F	8,47	8,15
COC1=C(NC(=O)C(=C)CN2CCOCC2)C=C3[N]4C=NC=C4C(=NC3=C1)NC5=CC=CC=C5C	6,53	6,94
CC1=C(NC2=NC3=CC=CC=C3[N]2C4=NC=NC(=C4)N)C=C(C=C1)C(=O)NC5=CC=CC6=CN=CC=C56	5,11	5,68
COC1=CC(=CC=C1NC(=O)C2=CC3=CC=CC=C3[N]2C)C4=CSC5=C(\C=C\CNC6CCNCC6)C=NC(=C45)N	5,43	5,50
COC1=CC=C(CN(C)C)C=C1C2=CC3=C(S2)N=CC(=C3NC4=C(C)C5=C([NH]C=C5)C=C4)C#N	7,64	6,39
CN(C)C/C=C/C(=O)N(C)C1=CC=C2N=C(NC3=CC(=CC=C3C)F)C4=CN=C[N]4C2=C1	5,86	6,21
CN(C)C/C=C/C(=O)N(C)C1=CC=C2N=C(NC3=C(O)C=CC=C3C)C4=CN=C[N]4C2=C1	6,78	6,83
CC1=C(NC2=NC3=CC=CC=C3[N]2C4=NC=NC(=C4)N)C=C(C=C1)C(=O)NC5=CC=CC(=C5)Br	6,57	6,34
CC1=CC=CC(=C1C2=CC3=NN=C(NC4=CC=C(OCCN5CCCC5)C=C4)N=C3C(=C2)C)C	7,68	7,85
CN(C)C/C=C/C(=O)N(C)C1=C(F)C=C2N=C(OC3=CC=C(F)C=C3C)C4=CN=C[N]4C2=C1	4,98	4,96
COC1=CC2=NC=CC(=C2C=C1OC)OC3=CC=C4C(=C3)C=CC=C4C(N)=O	7,06	7,15
CN(C)CC1=CC(=CC=C1)C2=CC3=C(S2)N=CC(=C3NC4=C(C)C5=C([NH]C=C5)C=C4)C#N	6,28	5,73
CN(C)C/C=C/C(=O)N(C)C1=CC=C2N=C(NC3=CC=CC(=C3C)Cl)C4=CN=C[N]4C2=C1	5,63	5,49
CC1=C(NC2=NC3=CC=CC=C3[N]2C4=NC=NC(=C4)N)C=C(NC(=O)C5=CC(=CC=C5)Cl)C=C1	6,88	6,52
CN(C)C/C=C/C(=O)N(C)C1=C(F)C=C2N=C(NC3=CC=CC=C3C)C4=CN=C[N]4C2=C1	6,45	6,60
CCOC1=CC=C(CN(C)C)C=C1C2=CC3=C(S2)N=CC(=C3NC4=C(C)C5=C([NH]C=C5)C=C4)C#N	7,13	6,38
CN(C)CC1=CC=C(C=C1)C2=CC3=C(S2)N=CC(=C3NC4=C(C)C5=C([NH]C=C5)C=C4)C#N	7,00	6,13

CNC1CC2OC(C)(C1OC)[N]3C4=C(C=CC=C4)C5=C6CNC(=O)C6=C7C8=C(C=CC=C8)[N]2C7=C35	8,89	8,42
CN(C)C/C=C/C(=O)N(C)C1=CC=C2N=C(NC3=CC=CC(=C3C)O)C4=CN=C[N]4C2=C1	6,31	6,36
CN(C)CC1=CC(=CC=C1)C2=CC3=C(S2)N=CC(=C3NC4=C(C)C5=C([NH]C=C5)C=C4)C#N	6,28	6,25
OC(=O)C1=CC=C(C=C1)C(=O)\C(SCC2=CC=C(Br)C=C2)=C/C3=CC=C(F)C(=C3)[N+]([O-])=O	4,77	5,19
CC1=C(NC2=NC3=CC=CC=C3[N]2C4=NC=NC(=C4)N)C=C(C=C1)C(=O)NC5=CC=CC(=C5)OC(F)F	7,00	7,09
CNC1=CC=C2N=C(OC3=CC=C(F)C=C3C)C4=CN=C[N]4C2=C1	6,25	6,68
O=C(CC1=CC=CC=C1)NC2=CC=CC(=C2)C3=C([N]4C=CSC4=N3)C5=CC=NC(=N5)NC6=CC=CC(=C6)N7CCOCC7	7,59	7,46
CC(C)(N)C(=O)NCC[N]1C=CC2=NC=NC(=C12)NC3=CC=C(OC4=CC=CC5=C4C=NS5)C(=C3)Cl	5,52	5,24
CC1=C(NC2=NC3=CC=CC=C3[N]2C4=NC=NC(=C4)N)C=C(NC(=O)C5=CC=C(S5)C(C)(C)C)C=C1	8,30	8,36
CN(C)CC1CCN(C1)CC2=C(C=C(C=C2)C(O)NC3=CC=C(C)C(=C3)NC4=NC(=CC=N4)C5=CN=CN=C5)C(F)(F)F	8,12	7,80
CNC1=CC=C2N=C(NC3=CC(=CC=C3C)F)C4=CN=C[N]4C2=C1	7,40	7,64
CCN(CC)CCCCNC1=CC(=NC=N1)[N]2C(=NC3=CC=CC=C23)NC4=C(C)C=CC(=C4)C(=O)NC5=CC(=CC=C5)C	5,88	5,61
CC1=CC(=CC=C1)NC(=O)C2=CC(=C(C)C=C2)NC3=NC4=CC=CC=C4[N]3C5=NC=NC(=C5)N	5,97	6,31
CN1CCN(CCCNC2=CC(=NC=N2)[N]3C(=NC4=CC=CC=C34)NC5=C(C)C=CC(=C5)C(=O)NC6=CC(=CC=C6)C)CC1	5,91	5,78
CC1=CC=CC(=C1)C(=O)NC2=CC(=C(C)C=C2)NC3=NC4=CC=CC=C4[N]3C5=NC=NC(=C5)N	7,09	6,35
NC1CCCCC1NC2=CC3=C(C(=O)NC=N3)C(=N2)NC4=CC=CC5=C4C=C[NH]5	7,49	7,13
CN1CCN(CC1)CC2=NC(=CC=C2)\C=C\C3=C(NC4=CC=C5[NH]C=CC5=C4C)C(=CN=C3)C#N	6,27	6,26
CC1=C(NC2=NC3=CC=CC=C3[N]2C4=NC=NC(=C4)NC5=CC=C(CCN6CCOCC6)C=N5)C(=CC=C1)Cl	6,66	7,54
NC1=C2C(=O)NC(=CC2=N[N]1C3=CC=CC=C3)C4=CC=CC=C4	5,85	5,91
OC1=CC(=CC=C1)NC2=CC=NC3=C2SC(=C3)C4=CC(=CC=C4)CN5CCOCC5	8,64	8,66
OC1CCN(CC1)C2=NC=CC(=C2)C3=CN=C4C=CC(=N[N]34)N5CCCC5C6=CC=CC(=C6)F	5,31	5,31
C[N]1C=C2C(=CC=CC2=N1)NC3=NC(=CC4=C3C(=O)NC=C4)NC5CCOCC5N	6,72	6,73
COC1=C(NC(=O)C#CCN2CCOCC2)C=C3[N]4C=NC=C4C(=NC3=C1)NC5=CC=CC=C5C	7,33	7,33
CN(C)C/C=C/C(=O)N(C)C1=CC=C2N=C(OC3=CC=C(F)C=C3C)C4=CN=C[N]4C2=C1	5,43	5,47
CN(C)C/C=C/C(=O)N(C)C1=CC=C2N=C(NC3=CC=CC(=C3C)F)C4=CN=C[N]4C2=C1	6,49	6,55
CC1=C(NC2=NC3=CC=CC=C3[N]2C4=NC=NC(=C4)NCCO)C=C(NC(=O)C5=CC=CC(=C5)C(F)(F)F)C=C1	6,40	6,17

CN(C)C/C=C/C(=O)N(C)C1=CC=C2N=C(NC3=CC(=CC=C3C)F)C4=CN=C[N]4C2=C1	5,85	5,85
CCN1CCN(CC1)CC2=CC=C(C=C2Cl)C(=O)NC3=CC(=C(C)C=C3)NC4=NC5=CC=CC=C5[N]4C6=NC=NC(=C6)N	8,00	8,00
COC(=O)C1=CC=C2C(C(=O)NC2=C1)C(=NC3=CC=C(C=C3)N(C)C(=O)CN4CCN(C)CC4)C5=CC=CC=C5	6,71	6,71
CC(C)(O)CC(=O)NCC[N]1C=CC2=C1C(=NC=N2)NC3=CC(=C(OC4=CC(=CC=C4)C(F)(F)F)C=C3)Cl	5,28	5,29
CC[N]1C=C(C2=CC=NC3=C2C=C[NH]3)C(=N1)C4=CC=C(NC(=O)NC5=CC=CC=C5)C=C4	6,40	6,41
CC1=C(NC2=NC3=CC=CC=C3[N]2C4=NC=NC(=C4)N)C=C(C=C1)C(=O)NC5=CC=CC=C5	5,26	5,25
CN1CCN(CC1)C2=CC=C(NC3=NC=C4C(=O)N(C5=NC6=CC=CC=C6[N]5C4=N3)C7=C(C)C=CC=C7C)C=C2	7,68	7,71
CC1=C[N](C=N1)C2=CC(=CC(=C2)NC(=O)C3=CC=C(C)C(=C3)NC4=NC(=CC=N4)C5=CN=CC=C5)C(F)(F)F	5,89	5,88
CC1=CC=C(C=C1)[N]2N=C(C=C2NC(=O)NC3=CC=C(C4=CN=C(CN5CCOCC5)C=C4)C6=CC=CC=C36)C(C)(C)C	6,00	5,99
CC1=C(C=C(NC(=O)C2=CC(=NC=C2)N3CCCCC3)C=C1)C4=CC=C(C=C4)C(=O)NCC5CC5	5,40	5,40
COC1=CC(=CC=C1NC(=O)C2=CC3=CC=CC=C3[N]2C)C4=CSC5=C(\C=C\CN6CCC(O)CC6)C=NC(=C45)N	5,28	5,26
NC1=C2C(=NC=N1)[N](N=C2C3=CC=C(OC4=CC=CC=C4)C=C3)C5CCCN(C5)C(=O)C=C	7,79	7,79
CC1=C(NC2=NC3=CC=CC=C3[N]2C4=NC=NC(=C4)NCCC#N)C=C(C=C1)C(=O)NC5=CC(=CC=C5)C(F)(F)F	6,33	6,33
NC1CCCCC1NC2=CC3=C(C(=O)NC=N3)C(=N2)NC4=CC=CC5=C4[NH]C=C5Cl	6,00	6,01
COC1=CC(=CC(=C1OC)OC)NC2=NC=C(F)C(=N2)NC3=NC4=C(OC(C)(C)C(=O)N4)C=C3	6,14	6,16
CNC1CC2OC(C)(C1OC)[N]3C4=C(C=CC=C4)C5=C6CNC(=O)C6=C7C8=CC=CC=C8[N]2C7=C35	9,31	9,31
CN(C)C/C=C/C(=O)N(C)C1=CC=C2N=C(NC3=CC=C(Cl)C=C3C)C4=CN=C[N]4C2=C1	6,42	6,56
CN(C)C/C=C/C(=O)N(C)C1=C(F)C=C2N=C(OC3=CC(=CC=C3C)F)C4=CN=C[N]4C2=C1	5,13	5,18
COC1=C(C=C2[N]3C=NC=C3C(=NC2=C1)NC4=CC=CC=C4C)N(CC5CC5)C(=O)\C=C\CN(C)C	5,70	5,71
C[N]1N=C(C=C1C(=O)NC2=CC(=C(C)C=C2)NC3=NC4=CC=CC=C4[N]3C5=NC=NC(=C5)N)C(C)(C)C	6,48	6,49
FC1=CC(=CC=C1)C2CCCN2C3=N[N]4C(=NC=C4C5=NC=CC=C5)C=C3	5,19	5,19
CN(C)C/C=C/C(=O)N(C)C1=C(Cl)C=C2N=C(NC3=CC=CC=C3C)C4=CN=C[N]4C2=C1	5,89	5,99
CN(C)C/C=C/C(=O)N(C)C1=CC=C2N=C(NC3=CC(=CC=C3C)Cl)C4=CN=C[N]4C2=C1	5,78	5,95
CC(C)(N)C(=O)NCC[N]1C=CC2=NC=NC(=C12)NC3=CC=C(OC4=CC=CC5=C4C=NS5)C(=C3)Cl	5,52	5,53
CCN(C(=O)\C=C\CN(C)C)C1=C(OC)C=C2N=C(NC3=CC=CC=C3C)C4=CN=C[N]4C2=C1	6,27	6,27
CCN1CCN(CC/C=C/C2=CC3=NC=C(C#N)C(=C3C=C2)NC4=CC(=C(SC5=NC=C[N]5C)C=C4)Cl)CC1	7,24	7,24

CN1CCN(CC1)CC2=C(Br)C=C(C=C2)C(=O)NC3=CC=C(C)C(=C3)NC4=NC(=CC=N4)C5=CN=CC=C5	8,51	8,51
CN(C)C/C=C/C(=O)N(C)C1=CC=C2N=C(SC3=CC=CC=C3C)C4=CN=C[N]4C2=C1	4,71	4,72
CN(C)CC1=CC(=CS1)C2=CC3=C(S2)N=CC(=C3NC4=C(C)C5=C([NH]C=C5)C=C4)C#N	6,39	6,35
CN(C)C/C=C/C(=O)N(C)C1=C(F)C=C2N=C(NC3=CC=CC=C3Cl)C4=CN=C[N]4C2=C1	5,67	5,69
CN1CCN(CCNC2=CC(=NC=N2)[N]3C(=NC4=CC=CC=C34)NC5=C(C)C=CC(=C5)C(=O)NC6=CC(=CC=C6)C)CC1	5,78	5,78
CN1CCC(CC1)NC2=CC(=NC=N2)[N]3C(=NC4=CC=CC=C34)NC5=C(C)C=CC(=C5)NC(=O)C6=CC=CC(=C6)C(F)(F)F	5,85	5,87
COC1=C(NC(=O)\C=C\CN(C)C)C=C2[N]3C=NC=C3C(=NC2=C1)NC4=CC=CC=C4C	7,96	7,97
CN(C)C/C=C/C(=O)N(C)C1=C(Cl)C=C2N=C(NC3=CC=C(F)C=C3C)C4=CN=C[N]4C2=C1	5,80	5,69
CC1=C(NC2=NC3=CC=CC=C3[N]2C4=NC=NC(=C4)N)C=C(NC(=O)C5=CC(=CC=C5)C(F)(F)F)C=C1	7,85	7,86
CC1=C(NC2=NC3=CC=CC=C3[N]2C4=NC=NC(=C4)NCCN)C=C(NC(=O)C5=CC=CC(=C5)C(F)(F)F)C=C1	5,83	6,03
FC(F)(F)C1=CC(=CC=C1)NC(=O)NC2=CC(=CC=C2)C3=C[N]4C=CN=C4C(=N3)NCC5=CC=NC=C5	5,22	5,23
CC1=C(NC2=NC3=CC=CC=C3[N]2C4=NC=NC(=C4)N)C=C(C=C1)C(=O)NC5=CC=CC(=C5)C(F)(F)F	7,82	7,83
COCCNC1=CC(=NC=N1)[N]2C(=NC3=CC=CC=C23)NC4=C(C)C=CC(=C4)NC(=O)C5=CC=CC(=C5)C(F)(F)F	6,90	6,93
OC1=C(C=C(Cl)C=C1)C(=O)NC2=CC=C(C=C2Cl)[N+]([O-])=O	4,62	4,64
O=C1NC=NC2=C1C(=NC(=C2)NC3CCCNC3)NC4=CC=CC5=C4[NH]C=C5	6,50	6,48
CN(C)C/C=C/C(=O)N(C)C1=CC=C2N=C(NC3=CC(=CC=C3C)O)C4=CN=C[N]4C2=C1	7,96	7,96
CN(C)C/C=C/C(=O)N(C)C1=C(F)C=C2N=C(OC3=CC=CC=C3C)C4=CN=C[N]4C2=C1	5,22	5,23
CC1=C(NC2=NC3=CC=CC=C3[N]2C4=NC=NC(=C4)NCCC5CCCN5)C=C(NC(=O)C6=CC=CC(=C6)C(F)(F)F)C=C1	6,09	6,11
NCC1=CC=C(C=C1)C2=CN=C3[NH]C=C(C4=CC=CC(=C4)NC(=O)NC5=C(F)C=C(C=C5)C(F)(F)F)C3=C2	7,40	7,43
CN(C)C/C=C/C(=O)N(C)C1=CC=C2N=C(NC3=C(Cl)C=CC=C3C)C4=CN=C[N]4C2=C1	7,35	6,85
